# Inhibition of Tapeworm Thioredoxin and Glutathione Pathways by an Oxadiazole *N-*Oxide Leads to Reduced *Mesocestoides* *vogae* Infection Burden in Mice

**DOI:** 10.3390/molecules200711793

**Published:** 2015-06-26

**Authors:** Vivian Pasquet, Hugo Bisio, Gloria V. López, Laura Romanelli-Cedrez, Mariana Bonilla, Jenny Saldaña, Gustavo Salinas

**Affiliations:** 1 Cátedra de Inmunología, Instituto de Higiene, Facultad de Química, Universidad de la República, Avda, A. Navarro 3051, Montevideo 11400, Uruguay; E-Mail: vivianpasquet@web.de; 2 Worm Biology Laboratory, Institut Pasteur Montevideo, Mataojo 2020, Montevideo 11400, Uruguay; E-Mails: bisioh@pasteur.edu.uy (H.B.); lromanelli@pasteur.edu.uy (L.R.-C.); 3 Grupo de Química Medicinal, Laboratorio de Química Orgánica, Facultad de Química-Facultad de Ciencias, Universidad de la República, Iguá 4225, Montevideo 11400, Uruguay; E-Mail: vlopez@fq.edu.uy; 4 Redox Biology of Trypanosomes Laboratory, Institut Pasteur Montevideo, Mataojo 2020, Montevideo 11400, Uruguay; E-Mail: mbonilla@pasteur.edu.uy; 5 Laboratorio de Experimentación Animal, Depto de Ciencias Farmacéuticas, Facultad de Química, Universidad de la República, Avda. Gral. Flores 2124, Montevideo 11800, Uruguay; E-Mail: jennysal@fq.edu.uy

**Keywords:** thioredoxin reductase, glutathione reductase, thioredoxin glutathione reductase, oxadiazole *N-*oxide, *Mesocestoides*, tapeworms

## Abstract

Parasitic flatworms cause serious infectious diseases that affect humans and livestock in vast regions of the world, yet there are few effective drugs to treat them. Thioredoxin glutathione reductase (TGR) is an essential enzyme for redox homeostasis in flatworm parasites and a promising pharmacological target. We purified to homogeneity and characterized the TGR from the tapeworm *Mesocestoides vogae* (syn. *M. corti*). This purification revealed absence of conventional TR and GR. The glutathione reductase activity of the purified TGR exhibits a hysteretic behavior typical of flatworm TGRs. Consistently, *M. vogae* genome analysis revealed the presence of a selenocysteine-containing TGR and absence of conventional TR and GR. *M. vogae* thioredoxin and glutathione reductase activities were inhibited by 3,4-bis(phenylsulfonyl)-1,2,5-oxadiazole *N*^2^-oxide (VL16E), an oxadiazole *N*-oxide previously identified as an inhibitor of fluke and tapeworm TGRs. Finally, we show that mice experimentally infected with *M. vogae* tetrathyridia and treated with either praziquantel, the reference drug for flatworm infections, or VL16E exhibited a 28% reduction of intraperitoneal larvae numbers compared to vehicle treated mice. Our results show that oxadiazole *N*-oxide is a promising chemotype *in vivo* and highlights the convenience of *M. vogae* as a model for rapid assessment of tapeworm infections *in vivo*.

## 1. Introduction

**Figure 1:**
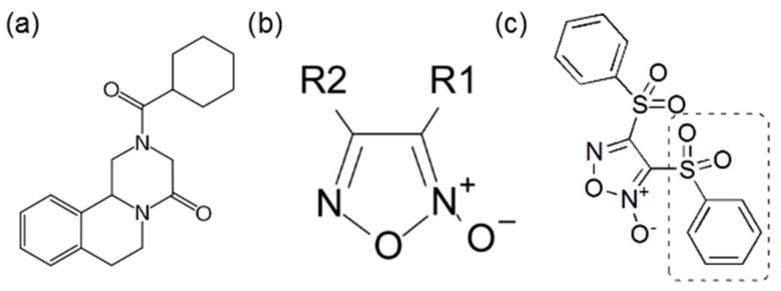
Molecular formula of (**a**) praziquantel; (**b**) a generic oxadiazole *N-*oxide and (**c**) 3,4-bis(phenylsulfonyl)-1,2,5-oxadiazole *N*^2^-oxide (VL16E). One of the phenylsulfonyl structural motifs of VL16E is marked.

Parasitic flatworms from two main classes, Cestoda (or tapeworms) and Trematoda (or flukes), cause chronic human and livestock infections and are a major cause of disability, mortality and significant economic losses in most developing countries [1,2]. There is no vaccine available for any human flatworm infection and large-scale treatment of flatworm infections relies on very few drugs [3,4,5]. A single drug, praziquantel (Figure 1a), is currently available for treatment of Schistosomasis, a flatworm disease that affects 200 million people worldwide. Benzimidazoles, like albendazole or mebendazole, are drugs partially effective for human infections caused by *Echinococcus* spp [6]. Furthermore, the emergence and spread of drug resistance against parasites is a serious issue nowadays [7,8,9]. The problem is aggravated since there is little research by pharmaceutical companies, due to the limited commercial interest of drugs that target flatworm infections. Thus, the identification of novel drugs remains as an important goal in public health, particularly in the poorest regions of the world. A rational target-based approach to the discovery of drug candidates holds promise to accelerate the process. A unique aspect of flatworm biochemistry is that these parasites are entirely dependent on the enzyme thioredoxin glutathione reductase (TGR, EC 1.8.1.B1) for the control of redox homeostasis and other essential processes. This enzyme functionally replaces both canonical thioredoxin reductase (EC 1.8.1.9) and glutathione reductase (EC 1.8.1.7) [10,11,12,13], and thus constitutes a redox bottleneck for flatworm metabolism. RNAi studies and assays with TGR specific inhibitors have shown that TGR is essential for the trematodes *Schistosoma mansoni* and *Schistosoma japonicum*, and validated this molecule as a novel drug target [14,15]. Subsequent high throughput screening of TGR inhibitors identified oxadiazole *N-*oxide (Figure 1b) as a drug hit for the control of schistosomiasis [16,17,18]. One of the oxadiazole *N*-oxides identified in this study partially cured *S. mansoni* experimental infection in mice [14]. TGR has also been found to be essential for the cestode flatworm *Echinococcus granulosus* [19]. It has been recently shown that oxadiazole *N-*oxides also inhibited *E. granulosus* TGR and killed both major classes of flatworm parasites: tapeworms and flukes [20]. That study showed that 3,4-bis(phenylsulfonyl)-1,2,5-oxadiazole *N*^2^-oxide (VL16E, Figure 1c) was among the most potent oxadiazole *N-*oxides tested and it was suggested that the phenylsulfonyl group of VL16E (Figure 1c) may be a potential chemotype in itself [20]. However, whether VL16E or other oxadiazole *N-*oxides are active *in vivo* against cestode infections has not been addressed yet. The lack of an established and convenient cestode model that allows cestocidal drugs to be tested in experimental infections has hampered research in this area. *Mesocestoides vogae* (*syn. corti*) [21,22], a rodent cestode parasite that does not infect humans has been proposed as an important *in vitro-in vivo* model to test and study cestocidal and anthelmintic drugs [23,24,25], due to its rapid mouse infection and its close relationship to cestodes of medical relevance, such as those from *Echinococcus* or *Taenia* genera. Here, we used *M. vogae* as a cestode experimental infection model to assess the efficacy of the previously identified TGR inhibitor VL16E. The results indicate that *M. vogae* thiol-redox network relies exclusively on a selenocysteine-containing TGR, a biochemical scenario similar to other flatworm parasites. We found that VL16E was as good as praziquantel in reducing asexual reproduction in *M. vogae* infection with tetrathyridial metacestodes. Our results highlight that *M. vogae* is a good model for flatworm parasite redox metabolism and provides further evidence that is a convenient model to examine cestocidal drugs *in vivo*.

## 2. Results and Discussion

### 2.1. Analysis of M. vogae Genome Revealed the Presence of One TGR Gene, and Absence of Conventional TR and GR Genes

**Figure 2:**
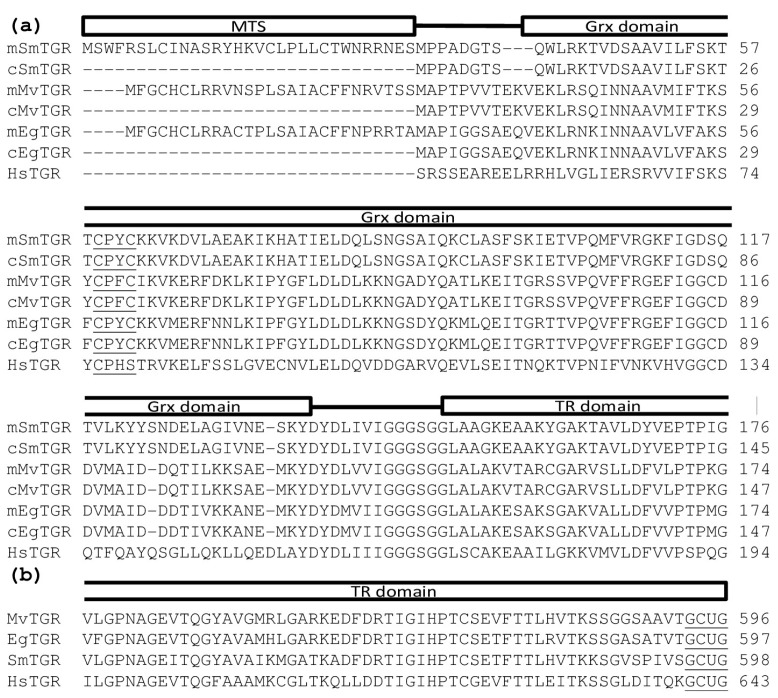
Protein sequence alignment of human and flatworm TGRs. Partial sequences from *M. vogae* (Mv), *S. mansoni* (Sm, AAK85233.1), *E. granulosus* (Eg, AAN63052.1) and *H. sapiens* (Hs, NP_443115.1) TGRs are aligned and N-terminus and C-terminus of the proteins are shown in (**a**,**b**), respectively. Conserved domains and mitochondrial targeting signal are represented by boxes above the alignment. Mitochondrial and cytosolic variants (m and c) for flatworm TGRs are shown. Grx domain active site and selenocysteine-containing TR domain are underlined.

*M. vogae* has recently been sequenced [26], however, the genes and proteins have not been annotated yet. Thus, we analyzed *M. vogae* genome for the presence of TGR and conventional TR and GR by tblastn using parasitic flatworm TGRs and conventional TR and GR from free-living flatworms as queries. This analysis revealed the presence of a TGR gene only. The deduced amino acid sequence indicates that *M. vogae* TGR contains an N-terminal glutaredoxin domain fused to a conventional thioredoxin reductase, similar to what has been observed for all other TGRs. The glutaredoxin domain contains a CX_2_C redox active center, typical of flatworm TGRs (Figure 2a). The TR domains contain a characteristic CX_4_C redox center and a C-terminal redox motif containing cysteine and selenocysteine (GCUG, where U denotes selenocysteine) (Figure 2a,b). The nucleotide sequence also revealed the existence of a selenocysteine insertion sequence element at the 3ʹUTR (data not shown) needed for recoding the UGA codon as selenocysteine. *M. vogae* TGR displays 81% identity to *E. granulosus* TGR. The genomic organization (number and size of exons and introns) of *M. vogae* TGR gene was very similar to the *E. granulosus* TGR gene. Interestingly, the gene model of *M. vogae* TGR predicts the existence of two transcript variants of TGR that would encode cytosolic and mitochondrial isoforms (Figure 2a). These variants would be identical in sequence except for an additional exon encoding a leader peptide that would give rise to the mitochondrial variant. The existence of two transcripts from a single TGR gene has been demonstrated for *E. granulosus* and *S. mansoni* [11]. 

### 2.2. M. vogae Thioredoxin Reductase and Glutathione Reductase Activities Co-Purified

**Figure 3:**
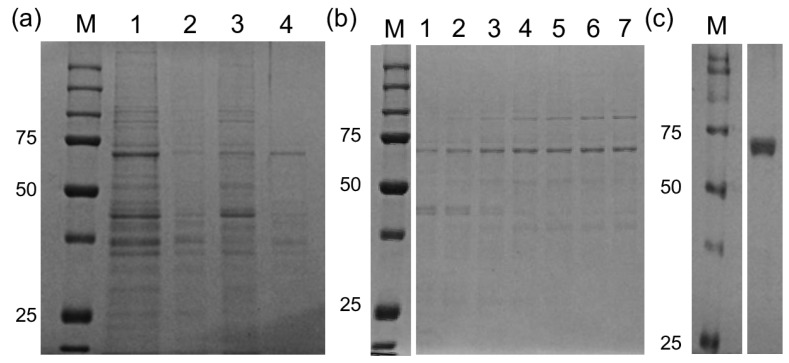
Purification of TGR from *M. vogae* tetrathyridium larvae. (**a**) Ammonium sulfate precipitation of a total aqueous extract from tetrathyridium larvae was carried out in three steps (0%–25%, 25%–50% and 50%–85% ammonium sulfate saturation) and analyzed by SDS-PAGE under reducing conditions (lane 1 corresponds to the total extract, lanes 2, 3 and 4 to 0%–25%, 25%–50% and 50%–85% ammonium sulfate saturation fractions, respectively); (**b**) The 25%–50% ammonium sulfate saturation fraction was further purified by size exclusion chromatography (SEC) on a 16/600 Superdex 200 column; lanes 1 to 7 show fractions were the TR and GR activities eluted; (**c**) SEC fractions containing TR and GR activities were pooled and subjected to a anionic chromatography on a MonoQ column. Bound proteins were eluted using a NaCl 10–1000 mM gradient and the fractions obtained were analyzed by SDS-PAGE under reducing conditions. The lane shown corresponds to the fraction with maximal TR and GR activity, which eluted at NaCl 300 mM. The gels shown in (**a**,**b**) were coomassie stained and the gel shown in (**c**) was silver stained. M denotes molecular weight markers.

Both TR and GR activities were traced in purification schemes from a total aqueous extract derived from 6 mL of packed *M. vogae* tetrathiridium larvae. In an optimized three step-wise ammonium sulfate fractionation (25%, 50%, 85% saturation solutions), 80% of both activities were recovered in the 25%–50% saturated ammonium sulfate fraction with approximately a two-fold enrichment (Figure 3a). Approximately 15% of TR and GR activities were recovered on the 50%–85% saturated ammonium sulfate fraction. The TR/GR activity ratio in these fractions was identical to the ratio of the initial total aqueous extract, suggesting the distribution of one enzyme in two fractions. The 25%–50% fraction was re-suspended, desalted and then chromatographed on a HiLoad 16/600 Superdex 200 PG. The size exclusion chromatography led to a nearly complete recovery (90%) of both activities and a 24-fold enrichment (Figure 3b). The GR and TR activities co-eluted in fractions corresponding to 120–140 kDa approximately, consistent with a dimeric holoprotein. The active fractions were pooled, desalted and chromatographed on anion exchange Mono Q column. Following anion exchange, a 68% recovery and a 25-fold enrichment were achieved (Figure 3c). An SDS-PAGE revealed the presence of a single band of approximately 65 kDa in the final step of purification (Figure 3c), consistent with the Mr of TGR. It is important to emphasize that both activities co-purified in all the purification steps. Furthermore, the same TR/GR activity ratio as the original extract was maintained in the purification process. Altogether, the data from the purification of native thioredoxin and glutathione reductases activities indicate that only TGR is responsible for both activities.

### 2.3. The GR Activity of M. vogae Exhibits Hysteresis towards Glutathione and is Inhibited by Auranofin

**Figure 4:**
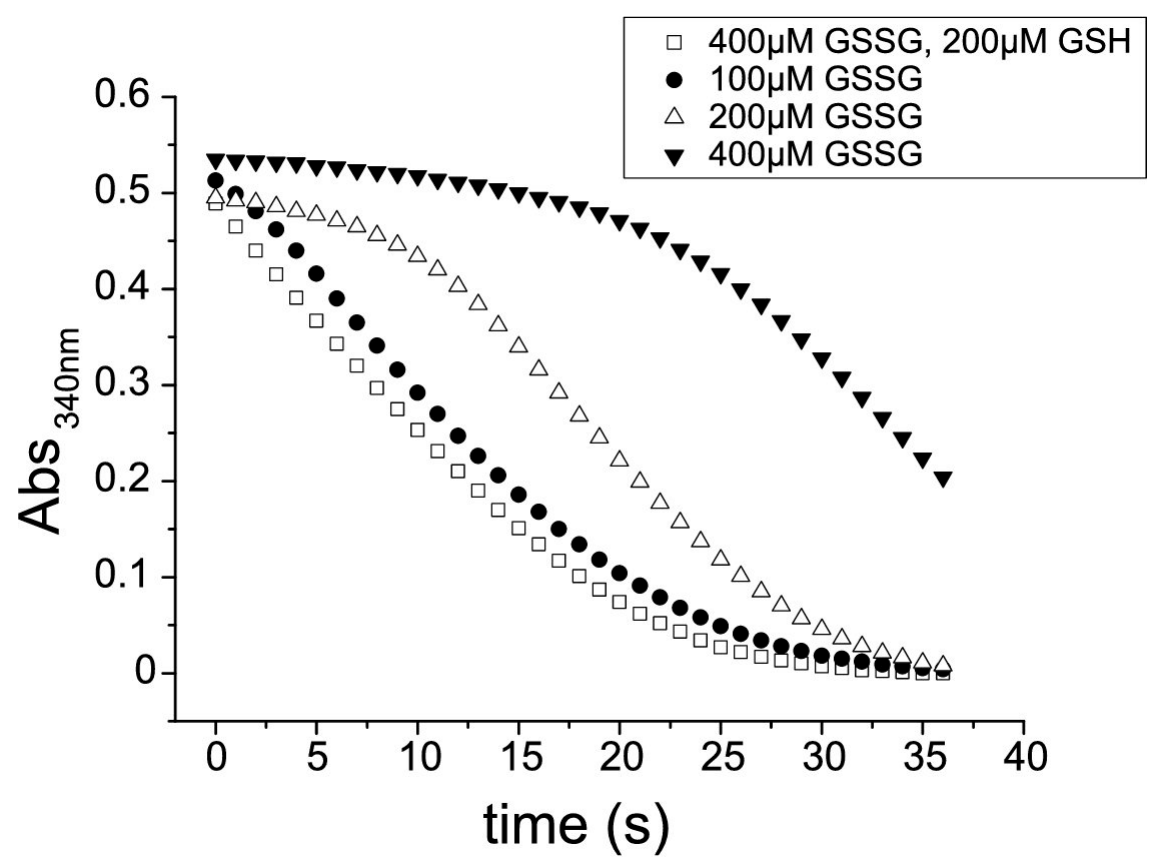
Hysteretic behavior of GR activity of TGR. Full-time courses obtained at different conditions are shown. In all cases constant nicotinamide adenine dinucleotide phosphate, reduced form (NADPH) and enzyme concentrations were used and reactions were started by the addition of enzyme. Oxidized glutathione (GSSG) and reduced glutathione (GSH) concentrations varied. GR assays were carried out with the purified TGR at a 2 nM concentration.

**Figure 5:**
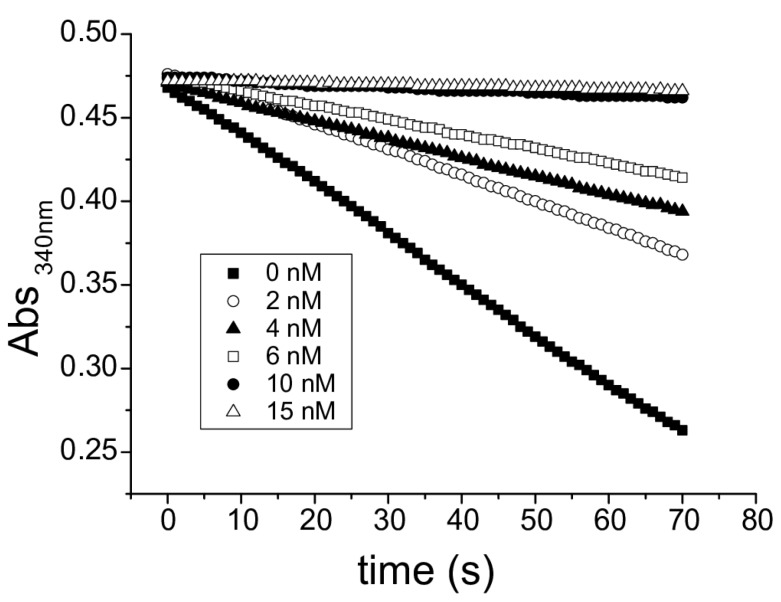
Inhibition of GR activity with Auranofin. Time-courses obtained using different auranofin concentrations are shown. GR assays were carried out at constant concentrations of TGR (2 nM), GSSG (50 µM), NADPH (100 µM) and without GSH. The enzyme was preincubated with the inhibitor during three minutes and the reaction was started by addition of GSSG.

A hysteretic behavior (*i.e.*, the existence of a lag time before full catalytic activity takes place) of GR activity at high concentration of oxidized glutathione is a footprint of flatworm TGRs and it has not been observed in canonical GR. Thus, we examined whether the GR activity of the purified enzyme exhibited hysteresis towards oxidized glutathione. A typical hysteretical behavior was observed: (i) hysteresis lag time increased as oxidized glutathione concentration increased; and (ii) the maximal slope of the curves did not change once the enzyme was fully active (Figure 4). Furthermore, addition of reduced glutathione to the GR assay mix abolished hysteresis (Figure 4), a conspicuous feature of TGR hysteretical behavior, which is controlled by the [GSSG]/[GSH] ratio [19,27,28]. Thus, these experiments provide further evidence that TGR is responsible for the GR activity in *M. vogae*. Auranofin is a known gold inhibitor of selenocysteine-containing TRs [29] and TGRs [10,14,15,27,30,31]. Both TR and GR activities present in the purified enzyme were inhibited by nanomolar concentrations of auranofin (Figure 5). Since conventional GRs do not possess selenocysteine at their active site, these results indicated that the GR activity is due to TGR and that selenocysteine is present at the TGR active site.

### 2.4. An Oxadiazole N-Oxide that Inhibits TGR Reduces M. vogae Infection Burden in Mice

**Figure 6:**
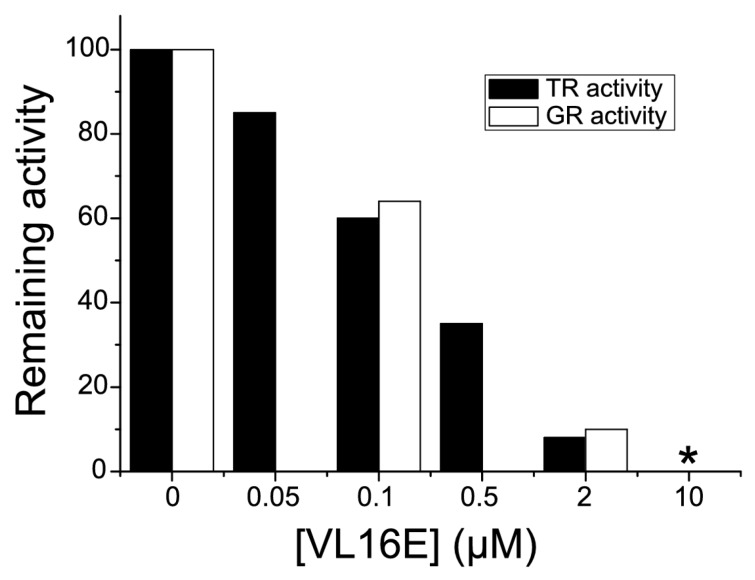
Inhibition of TR and GR activity of purified TGR from *M. vogae* by VL16E. Residual TR and GR activity as a function of inhibitor concentration is plotted in black and white respectively. Residual GR activity was measured only with 0.01 and 10 µM of inhibitor. (*)TR and GR activities could not be detected. TGR was used at 2nM concentration in all assays.

We have recently shown that 3,4-bis(phenylsulfonyl)-1,2,5-oxadiazole *N*^2^-oxide (VL16E, Figure 1c) is a potent TGR inhibitor that killed *in vitro* larval worms of the cestode parasite *E. granulosus* [20]. Whether VL16E is capable of cure infection in cestodes has not been addressed. *M. vogae* provides an excellent model to test this inhibitor *in vivo*. We first examined whether VL16E inhibits *M. vogae* TGR. Similar to the results obtained with *E. granulosus* TGR, the TR and GR activities of the purified TGR were inhibited by VL16E (Figure 6). Furthermore, VL16E killed tetrathyridium larvae *in vitro* at 20 µM concentration (data not shown).

**Figure 7:**
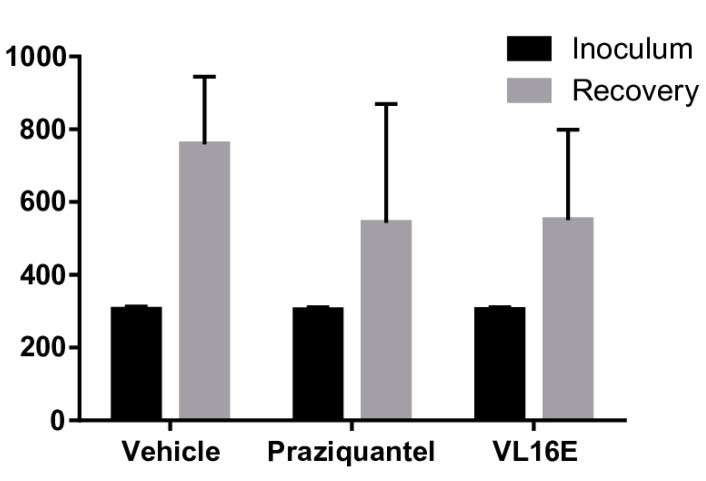
VL16E reduces infection burden of *M. vogae* infection in mice. Three groups of eight mice each were infected with approximately 300 *M. vogae* tetrathyridium larvae by intraperitoneal injection and treated by gavage at days 14, 15 and 16 after infection with vehicle (4% dimethyl sulfoxide in PBS, negative control group), praziquantel (positive control group) and VL16E (oxadiazole *N*-oxide 3,4-bis(phenylsulfonyl)-1,2,5-oxadiazole *N*^2^-oxide). At day 17 mice were sacrificed and the recovered larvae counted. Results are expressed as mean larval count and standard deviations. The increase in tetrathyridium count (recovery *vs.* inoculum) wss statistically significant in the vehicle group but not in treated groups (either praziquantel or VL16).

We then examined whether VL16E was active *in vivo* in *M. vogae* infection in mice. Three groups of mice (*n* = 8) were infected with *M. vogae* tetrathyridium. Infection was allowed to proceed for two weeks and treated during three consecutive days with either VL16E, a reference anthelmintic (praziquantel) or vehicle. The infection burden was assessed at day 17. The results are presented in Figure 7. A statistical analysis (non-parametric Mann-Whitney test) comparing inoculum and recovery within each group was performed. A significant 2.5-fold increase in parasite burden was observed in the vehicle control group. In contrast, only a 1.8-fold increase in parasite burden was observed in both groups treated with either praziquantel or VL16E for three days. A statistical analysis comparing inoculum and recovery within each group indicated that the increase in parasite burden was significant in the control group but not in the treated groups. A 28.5% and 28% reduction in tetrathyridium recovery was observed in the groups treated with praziquantel and VL16E, respectively, compared to tetrathyridium recovery in the vehicle control group (Figure 7). Although under the conditions assayed, VL16E performed equally well to praziquantel, the drug of choice for several flatworm infections, the decrease in parasite burden was not statistically supported using an α = 0.05 (non-parametric test Kruskal-Wallis). Importantly, no evident side effects were observed in mice during treatment with VL16E.

### 2.5. Discussion

The redox systems of several parasitic flatworms are unique in that conventional TR and GR have been replaced by TGR. TGR is currently one of the most attractive drug targets for flatworm infections, since it is an essential and “druggable” enzyme whose inhibition leads to redox homeostasis disruption and death of flatworm parasites [14,15,20,32]. However, it is not known whether this pathway setup is common to all parasitic flatworms, particularly considering that free-living flatworms have TR, GR and TGR [11]. In this article, we demonstrate that the cestode *M. vogae* shares the same biochemical scenario as other parasitic flatworms regarding thioredoxin and glutathione pathways. The results are clearly indicative of the existence of TGR, and absence of conventional TR and GR. The genome mining of *M. vogae*, which has recently been published, but not annotated, indicated that this parasite lacks TR and GR genes and possesses a selenocysteine-containing TGR gene. The selenocysteine residue at the prototypical carboxy terminal GCUG redox center is coded by an in frame UGA codon and the presence of a SECIS element in the 3ʹUTR allows UGA to be recoded as selenocysteine. Furthermore, the predicted domains of *M. vogae* TGR are identical to that of other TGRs; with a C-terminal TR module that would be responsible for the TR activity and an N-terminal Grx domain that, in combination with the TR module would be responsible for the GR activity. We purified to homogeneity the TR and GR activities from *M. vogae* extracts. Both enzymatic activities co-purified at a constant GR/TR ratio in the purification scheme. After salting in, size exclusion chromatography and ion exchange, a single protein band was isolated and its size corresponded to the molecular weight of TGR (65 kDa). No bands corresponding to conventional TR (54 kDa) or conventional GR (52 kDa) were observed, in full agreement with the genome data mining. Furthermore, the biochemical characterization revealed that the purified activities correspond, in all likelihood to TGR. The hysteretical behavior of the GR activity observed at high concentrations of oxidized glutathione (Figure 4) is an “enzymatic signature” of TGRs that contain a dithiolic redox active site at the N-terminal glutaredoxin domain, as the one deduced from *M. vogae* TGR gene. Indeed this phenomenon is not observed in conventional GR and, it is a conspicuous feature so far observed exclusively in flatworm TGRs [19,27,28,33,34]. Finally, the inhibition of *M. vogae* TGR with auranofin affected both GR and TR activities indicating that the purified TGR contains selenocysteine at its redox active site. These results highlight that *M. vogae* is a particularly relevant model to test compounds that target the thioredoxin and glutathione pathways, since this parasite shares the same metabolism with other parasitic flatworms. Furthermore, TGR inhibitors will target cytosolic and mitochondrial redox homeostasis: similar to other flatworms, the TGR gene organization predicts the existence of two TGR variants, cytosolic and mitochondrial, derived from the same gene and differing exclusively in the presence of an additional leader peptide in the mitochondrial isoform.

The TGR inhibitors oxadiazole *N-*oxides have been shown to be a potential drug hit for flatworm infections and have been shown to be effective in treating trematode (fluke) experimental infections [16]. We have previously described that oxadiazoles were able to kill the cestode *E. granulosus* larval worms *in vitro* [19]. However, whether oxadiazole *N-*oxides are effective *in vivo* against cestodes (tapeworms), the other major class of parasitic flatworms, had not been assessed yet. Testing *E. granulosus* experimental infection in mice takes at least nine months [35] and assessment of infection by cyst number and size is not reliable due to low numbers and variability in infection rates. Thus, we addressed this issue by using *M. vogae* as a cestode model for infections. *M vogae* is a convenient cestode parasite to assess potential drug hits *in vivo*. *M. vogae* possesses an unparalleled proliferation rate of its larval form, tetrathyridium, in experimental infections in mice, and therefore allows parasite growth rate to be tested in two weeks. We tested the cestocidal activity of oxadiazole *N*-oxide 3,4-bis(phenylsulfonyl)-1,2,5-oxadiazole *N*^2^-oxide (VL16E) since it was one of the most potent inhibitors *in vitro* and because the phenylsulfonyl moiety was suggested as an important determinant for the activity. Before testing VL16E in infection studies, we show that VL16E inhibited both thioredoxin and glutathione reductase activities of the purified *M. vogae* TGR. We then examined whether VL16E cured *M. vogae* infection in mice. In parallel to the vehicle control group and VL16E, we used a control group with praziquantel, the reference drug for cestodes. The results obtained indicated that after two weeks of infection and three days of treatment, the increase in tetrathiridium larval count during infection was statistically significant (*p* ≤ 0.05) in the vehicle control group, but not in the VL16E or praziquantel treated groups. In other words, the analysis of inoculum *vs.* recovery within each group revealed that only the non-treated group showed a statistically significant increase in parasite burden. Infection recoveries were very similar in the groups treated with either drugs, and showed a 28% average reduction of parasitaemia in comparison to the vehicle control group. Although this reduction in parasite recovery was not statistically supported the results are promising. It is important to highlight that the most strict positive control group was included in the experimental design (*i.e.*, praziquantel). It should also be noted that this effect is associated with a three-day treatment only. The doses of either drug used in the present study were similar to those used in other studies [14,16]. Importantly, VL16E was administered by gavage, indicating that VL16E is active by this route. It is also relevant to highlight that no notorious side effects were observed in the mice group treated with VL16E. Thus, our results showed a good correlation of TGR inhibition, *in vitro* and *in vivo* antiparasitic activity for VL16E. Overall, these results reinforce the idea that oxadiazole *N-*oxides are promising chemotype for flatworm infections that deserve to be further explored. This is particularly relevant considering that flatworm infections are chronic and debilitating diseases and there is a very restricted set of drugs and a few rational drug targets and supports TGR as a valid target in the development of drugs against tapeworm and fluke parasites. 

## 3. Experimental Section

### 3.1. Mesocestoides vogae Tetrathyridia Infection

Two months old CD1 mice were infected with fresh *M. vogae* tetrathyridia in saline solution (0.3 mL, *ca*. 300 tetrathyridia) by intraperitoneal inoculation, according to [25,36]. Infection was allowed to proceed for a variable time and then the mice were killed by cervical dislocation. The animal protocol complied with Uruguayan Law No. 18.611 [37] and it was harmonized with The Canadian Guidelines on Animal Care. Experimental protocol n 02-05-10 of the study was reviewed and approved by IACUC of Facultad de Química, Universidad de la República, Uruguay.

### 3.2. Enzyme Purification from Mesocestoides vogae

Tetrathyridia larvae were collected from the peritoneum of three month infected mice, washed by sedimentation with a saline solution and frozen at −80 °C until use. Six mL of tetrathyridia larvae were grounded to a powder under liquid nitrogen using a mortar and a pestle. Five mL of phosphate saline buffer (PBS) pH 7.6, containing 50 mM NaH_2_PO_4_, 100 mM NaCl, 0.05% tween, 1 mM EDTA, 1 mM PMSF and 100 µg/mL DNAse, were added to the thawed suspension and sonicated. The suspension obtained was centrifuged at 20,000 *g* during 30 min and the supernatant containing the aqueous protein extract was used for purification. Ammonium sulfate precipitation of the extract was carried out in three steps of 25%, 50% and 85% saturation. Most of the thioredoxin reductase (TR) and glutathione reductase (GR) activities (see enzymatic assays below) were present in the 25%–50% fraction; less than 10% of TR and GR activities were present in the 50%–85% fraction. The 25%–50% fraction was re-suspended in water and immediately dialyzed against PBS and applied to a HiLoad 16/600 Superdex 200 pg (GE Healthcare Life Sciences, Buckinghamshire, UK). All fractions were analyzed for TR and GR activities. Fractions containing TR and GR activities were pooled and dialyzed against Tris 20 mM, NaCl 10 mM, pH 8. The dialyzed fraction was applied to a MonoQ™ 5/50 GL column (GE Healthcare Life Sciences) previously equilibrated in Tris 20 mM, NaCl 10 mM, pH 8. After washing with equilibration buffer, the retained material was eluted by a salt concentration gradient (10–1000 mM NaCl in 15 column volumes). Enzyme activities were measured in the unbound and bound fractions. The fractions were analyzed in 10% SDS-PAGE gels under reducing conditions according to standard techniques. Gels were coomassie or silver stained (the latter staining for the final purification step). Precision plus Protein™ (BIO_RAD) molecular weight markers were used. Protein concentration was determined using Bradford reagent [38,39].

### 3.3. DTNB Reduction Assay for TR Activity

The reduction of 5,5ʹ-dithiobis(2-dinitrobenzoic acid) (DTNB) with concomitant NADPH oxidation was determined by the increase in absorbance at 412 nm due to the formation of 5ʹ-thionitrobenzoic acid (TNB) (ε = 13,600 M^−1^·cm^−1^) [40]. The reaction mixtures contained 100 μM NADPH and 5 mM DTNB in phosphate buffer 50 mM, EDTA 1 mM, pH 7. The reaction was followed for three minutes. Control experiments without NADPH were performed.

### 3.4. GSSG Reduction Assay

The GR activity was assayed as the NADPH-dependent reduction of oxidized glutathione (GSSG), which is followed by the decrease in absorbance at 340 nm due to NADPH oxidation (ε = 6200 M^−1^·cm^−1^) [41]. The reaction mixtures contained 100 μM NADPH, 1 mM GSSG and 1 mM GSH to avoid conditions of hysteresis in phosphate buffer 50 mM, EDTA 1 mM, pH 7 [19,27,28,33]. The hysteresis of glutathione reductase was studied by varying the concentration of GSH from 0 to 1 mM GSH in the assay. The reaction was recorded until depletion of NADPH.

### 3.5. Inhibition Studies

In all cases, fractions containing TGR were preincubated during three minutes with NADPH and the inhibitor VL16E or auranofin, a specific inhibitor of selenocysteine-containing TRs and TGRs. The reaction was started by the addition of DTNB for thioredoxin reductase assay or GSSG for glutathione reductase assay (see above). All assays were performed in triplicate. In every case, controls for reaction without enzyme and without inhibitor were carried out in parallel. The residual TR or GR activity after inhibition was calculated as: (v_i_/v_o_) × 100, where v_i_ and v_o_ correspond to the initial velocities of TNB formation (TR assay) or NADPH consumption (GR assay) with and without inhibitor, respectively. A range of inhibitor concentrations from 10 nm to 10 µM was assayed. 

### 3.6. Cestocidal Effect of VL16E in Tetrathyridia Infected Mice

Cestocidal effect of VL16E was tested using mice infected by the same procedure described above. Three groups of mice (eight mice each) were separated and treated with VL16E, the reference drug praziquantel or vehicle (4% dimethyl sulfoxide in PBS). On days 14, 15 and 16 after infection, mice received a daily dose of praziquantel (20 mg/kg body weight in a 0.2 mL volume), VL16E (equimolar dose to praziquantel, in a 0.2 mL volume) or vehicle (0.2 mL) administered by gavage. At day 17, post-infection, the animals were killed by cervical dislocation and the larvae collected from their peritoneum, washed and counted. Parasite recovery (larval count) from control and treated groups was determined and the efficacy of chemotherapy estimated. The efficacy of chemotherapy was estimated through the percentage of reduction tetrathyridium recovery, calculated as: % reduction = 100 × (mean from control group − mean from treated group)/mean from control group (where mean refers to the mean number of recovered parasites). The differences between the mean number of recovered and inoculated parasites in each group were tested for statistical significance using the non-parametric Mann-Whitney test (α = 0.05). The statistical analysis to compare parasite recovery among the three groups was performed using the non-parametric test Kruskal-Wallis.

### 3.7. Sequence Analysis

In order to obtain information on putative TR, GR and TGR sequences from *M vogae* the 50 Helminth Genomes Initiative database [26] was searched by tblastn using the amino acid sequences of TGR from *E. granulosus* (a parasitic flatworm) and GR and TR from *Schmidtea mediterranea* (a free-living flatworm) as queries. Since proteins and gene models have not been predicted for *M. vogae*, the coding sequence of *M. vogae* TGR was mapped using the exon and intron information of *E. granulosus* and *S. mansoni* TGRs. Final adjustments of intron-exon boundaries were performed based on a multiple alignment of TGRs from *E. granulosus Echinococcuus multilocularis*, *Hymenolepis microstoma* and *Taenia solium*. The multiple alignment of flatworm TGRs was carried out using ClustalW2 [42]. The selenocysteine insertion sequence (SECIS) elements present in *M. vogae* TGR was identified using the SECISearch program, version 2-19 [43,44] using the default energy cutoffs and the canonical pattern. Topology prediction of the polypeptides was carried out using signal P [45] and ipSORT [46].
